# Effect of Different Disinfection Protocols on Microbial and Biofilm Contamination of Dental Unit Waterlines in Community Dental Practices

**DOI:** 10.3390/ijerph110202064

**Published:** 2014-02-18

**Authors:** Laura Dallolio, Amalia Scuderi, Maria S. Rini, Sabrina Valente, Patrizia Farruggia, Maria A. Bucci Sabattini, Gianandrea Pasquinelli, Anna Acacci, Greta Roncarati, Erica Leoni

**Affiliations:** 1 Unit of Hygiene, Public Health and Medical Statistics, Department of Biomedical and Neuromotor Sciences, University of Bologna, via S. Giacomo 12, Bologna 40126, Italy; E-Mail: erica.leoni@unibo.it; 2 Unit of Hygiene and Quality of Residencial Services, Bologna Local Health Authority, Bellaria Hospital, via Altura 3, Bologna 40139, Italy; E-Mails: amalia.scuderi@ausl.bologna.it (A.S.); mariasofia.rini@ausl.bologna.it (M.S.R.); patrizia.farruggia@ausl.bologna.it (P.F.); anna.acacci@ausl.bologna.it (A.A.); greta.roncarati@ausl.bologna.it (G.R.); 3 Department of Specialized, Experimental, and Diagnostic Medicine, University of Bologna, via Massarenti 9, Bologna 40138, Italy; E-Mails: sabrina.valente2@unibo.it (S.V.); gianandr.pasquinelli@unibo.it (G.P.); 4 Bologna Provincial Division, Agency for Environmental Protection and Health Prevention in Emilia-Romagna, via Triachini 17, Bologna 40138, Italy; E-Mail: mbucci@arpa.emr.it

**Keywords:** dental unit waterlines, water disinfection, biofilm, peracetic acid, hydrogen peroxide, chlorine dioxide

## Abstract

Output water from dental unit waterlines (DUWLs) may be a potential source of infection for both dental healthcare staff and patients. This study compared the efficacy of different disinfection methods with regard to the water quality and the presence of biofilm in DUWLs. Five dental units operating in a public dental health care setting were selected. The control dental unit had no disinfection system; two were disinfected intermittently with peracetic acid/hydrogen peroxide 0.26% and two underwent continuous disinfection with hydrogen peroxide/silver ions (0.02%) and stabilized chlorine dioxide (0.22%), respectively. After three months of applying the disinfection protocols, continuous disinfection systems were more effective than intermittent systems in reducing the microbial contamination of the water, allowing compliance with the CDC guidelines and the European Council regulatory thresholds for drinking water. *P. aeruginosa*, *Legionella* spp, sulphite-reducing *Clostridium* spores, *S. aureus* and β-haemolytic streptococci were also absent from units treated with continuous disinfection. The biofilm covering the DUWLs was more extensive, thicker and more friable in the intermittent disinfection dental units than in those with continuous disinfection. Overall, the findings showed that the products used for continuous disinfection of dental unit waterlines showed statistically better results than the intermittent treatment products under the study conditions.

## 1. Introduction

Output water from dental unit waterlines (DUWLs) may be a potential source of infection for both dental health care personnel and patients. Many studies have shown that the output water of dental units is colonized with microorganisms including environmental bacteria, opportunistic and true human pathogens and organisms commonly found in the oral cavity (e.g., Streptococci) [1,2,3,4,5,6], suggesting that bacteria may originate from both incoming tap water and from patients by suck-back. Contamination by retrograde aspiration of oral secretions, more frequently observed in the past, has been greatly reduced by the incorporation in the handpieces of anti-retraction valves, which prevent suck-back of oral microbes and hence reduce the risk of contamination from this source [3,7]. The presence of bacteria within the tubing is conducive to the formation of biofilm that becomes the primary reservoir for continuous contamination of the system [7,8,9].

Although data on healthcare-associated infections in dentistry are under-reported in the literature [10], a small number of case-reports directly linked to contaminated DUWLs are described. A recent fatal case of pneumonia due to *Legionella* was reported in an Italian 82-year-old woman [11]. A single case of prosthetic heart valve infection due to *Micobacterium gordonae* [12] and two cases of cervical lymphadenitis caused by non-tuberculous *Mycobacterium* spp were also reported [13]. Other possible but not definitively proved infections were two cases of oral abscesses by *P. aeruginosa* in patients with cancer [14] and a case of amoebic keratitis in a contact lens wearer [15]. Finally, there are no reports of occupational infections associated with contaminated DUWLs in dental healthcare staff, but there is evidence for adverse health effects on dentists following exposure to bacterial endotoxins in dental unit output water [16] and a high prevalence of *Legionella* seropositivity was observed in dental personnel [17]. Although there is no epidemiological evidence pointing to a public health problem [18], the presence of a substantial number of pathogens is cause for concern due to the increasing number of vulnerable individuals, e.g., the elderly, immune-compromised individuals, and because of the adoption of increasingly sophisticated procedures and technologies. Moreover, exposing patients or dental health care personnel to water of uncertain microbiological quality, despite the lack of documented effects with significant impact on public health, is inconsistent with accepted infection control principles [18,19].

The guidelines of the Centers for Disease Control (CDC) for dental healthcare settings recommend that the water used for routine dental treatment meet regulatory standards for drinking water (≤500 CFU/mL of heterotrophic water bacteria) established by Environmental Protection Agency (EPA) [18,20]. In Italy standards for drinking water are more restrictive and include: Heterotrophic Plate Count at 22 °C ≤ 100 CFU/mL, Heterotrophic Plate Count at 36 °C ≤ 20 CFU/mL and the total absence/100 mL of *P. aeruginosa*, according to the European Council Directive 98/83 [21,22].

An integrated approach has been suggested in order to adhere to these limits, which includes waterline flushing, independent water reservoir systems, distilled or sterilized water, inline micro pore filtration, anti-retraction valves, periodic or continuous chemical disinfection and adherence to disinfection protocols. A wide range of chemical disinfectants have been studied for use in DUWLs: hydrogen peroxide [23,24], hydrogen peroxide with silver ions [19,25,26], chlorine dioxide [27,28], peracetic acid [29], sodium hypochlorite [30] and chlorhexidine gluconate [19]. The efficacy of different DUWL chemical treatments has been compared in a significant number of studies [8,30,31], but only few have been conducted in a dental practice setting [7,19,29]. 

The purpose of this study was to carry out a comparative evaluation of the efficacy of different disinfection protocols with regard to the microbiological quality of the output water from dental units and the presence of biofilm in DUWL air-water syringes and turbines. The investigation was carried out in community dental care centers, during routine practice. Due to their characteristic of being run by the public health system and offering their services free or at low cost, a high percentage of patients come from the less wealthy population (immigrants, the elderly, drug addicts, transients) who on account of their socio-sanitary conditions may be more prone to infections.

## 2. Materials and Methods

### 2.1. Dental Units

The five dental units tested were in operation in community clinics of the Italian National Health Service in a city in northern Italy and were comparable in terms of work load, type of activity and age (3–4 years). In the period previous to the study the municipal water was the mains source water for the dental units and a waterline disinfection protocol was not in use. The mains water was tested at the beginning and at the end of the study and always met the Italian drinking water standards. In accordance with Local Health Authority protocols, the microbiological content of dental unit output water was previously measured every six months and no significant differences had been observed in the input and output water of the various units selected. At baseline, in order to minimize differences between dental units, a cycle of disinfection with hydrogen peroxide was carried out, by entering 250 mL of hydrogen peroxide 3%. After 10 min of contact, the waterlines were washed with the supply water flushing under normal operating conditions. One of the dental units was then used as a control, two had intermittent disinfection and two had continuous disinfection. The control unit continued to be supplied with municipal water and underwent no further disinfection. In the intermittent disinfection, a peroxidic system was tested, in equilibrium at pH 8, which generates peracetic acid, peracetyl ions and hydrogen peroxide equivalent to 0.26% of peracetic acid (PeraSafe^®^, Antec International, Sudbury, Suffolk, UK) In accordance with the manufacturer recommendation, the intermittent disinfection procedure was implemented before treating each patient [19]. The dental care worker was instructed to achieve a between-patient disinfection cycle which consists in an automated dosage of the product into the dental unit waterlines for a contact time of 10 minutes, followed by a final flushing with supply water for 5 min. In the continuous disinfection a dosing pump was set up to add the disinfectants continuously to the waterlines. The products tested were hydrogen peroxide 0.02% plus silver ions (Oxygenal6, KaVo Dental GmbH, Biberach, Germany,) applied to deionized water and stabilized ClO_2_ 0.22% (Osmosteril Attila^®^, Ecoplus srl, Bologna, Italy) applied to water subjected to reverse osmosis. When the units were not in use no treatment was applied. In order to minimize any differences between the dental workers, the staff were appropriately and continuously trained in the methods and good practices to be adopted. 

### 2.2. Collection and Processing of Water Samples

Approximately three months after the implementation of the disinfection protocols, samples were collected from each unit for three consecutive weeks, two days a week and three times a day (at the beginning, in the middle, and at the end of the workday) for a total of 90 water samples, 18 for each dental unit. Before taking the samples from the distal outlets of the air/water syringes and turbines, water was flushed for two minutes. In the units undergoing intermittent treatment, water samples were collected when the disinfection cycle was not in action. In order to neutralize the residual disinfectant in water with continuous treatment, 10% sodium thiosulphate was added in sterile bottles for bacteriological analysis (1 mL·L^−^^1^). The samples were kept at 4 °C and analyzed within 24 h of sampling.

The following process indicators were chosen: the total Heterotrophic Plate count at 22 °C (HPC 22 °C), the total Heterotrophic Plate count at 36 °C (HPC 36 °C), *P*. *aeruginosa*, *Legionella* spp., and sulphite-reducing *Clostridium* spores. Human indicators were also chosen: *Staphylococcus aureus* and β-haemolytic streptococci. The HPCs were performed by pour plate method on Plate Count Agar (Italian Biolife, Milan, Italy) at 36 °C and 22 °C, for 48 and 72 h respectively [21,22,32]. *Legionella* spp. was detected according to the ISO11731 standard technique (1998) by pouring 1 liter of water through a nylon filter with 0.22 µm diameter pores (Millipore, Bedford, MA, USA). The concentrate was suspended in 10 mL of sample water and vortexed for 15 min. An aliquot of the concentrate was examined as such, another aliquot was subjected to decontamination treatment with heat at 50 °C for 30 min. Both the concentrated and decontaminated samples were plated on Legionella MWY selective Agar (Oxoid, Basingstoke, UK) and incubated at 35 °C in microaerophilic conditions for 14 days. The isolates were identified on the basis of cultural and serological features, as previously described [33,34]. The standard filtering membrane technique (APHA, 2005) was used to detect *S. aureus* on Baird Parker Agar (Biolife), β-Hemolytic Streptococci on Columbia CNA-CV Blood Agar (Biolife), *P. aeruginosa* on Pseudomonas Selective Agar (Biolife) and sulphite-reducing *Clostridium* spores on Sulfite Agar A (BBL-Difco BD, Sparks, MD, USA) [32]. For each test a volume of 100 mL was filtered using cellulose acetate filters with a porosity of 0.45 µm (Millipore). After an incubation period at 36 °C for 48–72 h (in anaerobiosis for *Clostridium*), suspected colonies were identified using miniaturized biochemical tests (API, bioMérieux, Marcy l’Etoile, France).

### 2.3. Biofilm

The PVC tubes feeding the air-water syringes and turbines were aseptically cut and collected at the beginning of the disinfection protocols (T_0_) and at the end of the study, after around three months of treatment (T_1_). Surface analysis of the air-water syringes and turbine tubes was performed using scanning electron microscopy (SEM). 15 mm-length tube samples were analyzed. Each sample was cut longitudinally into four parts with a razor blade and glued onto aluminium stubs using silver paste. The samples were then coated with a 10 nm thick gold film in a Balzers MED 010 sputtering device (Balzers, Hudson, NH, USA) and examined under a Philips 505 scanning electron microscope (FEI, Hillsboro, OR, USA) at 10–15 kV.

Semiquantitative SEM analysis included the evaluation of (a) the presence or absence of surface biofilm and its average thickness; (b) the friability of biofilm; and (c) the presence or absence of bulk bulging deposits. Biofilm detection was performed at one hundred times magnification on at least twenty consecutive fields. The average thickness of the biofilm was determined by digital images taken at 1,500× and measured using ImageProPlus software (MediaCybernetics) [35]. Biofilm friability and the presence of bulk deposits were evaluated at the same surface sites. The results were expressed as the percentage of surface examined that presented biofilm in different thicknesses (<1 µm; 1–3 µm; >3 µm), phenomena of detachment and fragmentation (friability), and bulk bulging aggregates. 

### 2.4. Statistical Analysis

All statistical analyses were performed using the SPSS program 20.0 publisher (IBM, Armonk, NY, USA). The values of microbial loads were converted into Log_10_ x to normalize the non normal distributions, and results are presented as geometric means. For negative samples, the detection limit was used. Differences between groups were tested using standard one-way analysis of variance (ANOVA). Categorical data about biofilm were analyzed with between-group comparisons using the chi-square or Fisher exact test, as appropriate. A *p* value ≤ 0.05 was considered statistically significant.

## 3. Results and Discussion

### 3.1. Water Samples

At baseline, immediately after the preliminary treatment with hydrogen peroxide, total hetrotrophic bacteria were present at low concentrations (HPC 22 °C and 36 °C < 5 CFU/mL) and the other tested microorganisms were not detected in all the samples. Table 1 shows the HPC patterns at 22 °C and 36 °C and the contamination from *P. aeruginosa* in the water taken from the dental units, in relation to the disinfection protocol implemented in the next 3–4 months. *Legionella* spp, sulphite-reducing *Clostridium* spores, *S. aureus* and β-haemolytic streptococci were never detected in any of the dental units. Means and standard deviations of microbial loads were calculated with no distinction between the different times of the day, since none of the units showed any difference in microbial loads for the three daily sampling times (ANOVA repeated measures, *p* > 0.05). 

**Table 1:** Microbial contamination of dental unit waterlines.

	Control	Intermittent Disinfection		Continuous Disinfection	
Microbiological indicators	untreated	hydrogen peroxide, peracetic acid 0.26%	hydrogen peroxide, peracetic acid 0.26%	hydrogen peroxide, silver ions 0.02%	stabilized ClO_2_ 0.22%
	mains water n: 18	mains water n: 18	deionized water n: 18	deionized water n: 18	reverse osmosis n: 18
**HPC 22 °C**					
positive samples (%)	100%	100%	100%	22.2%	55.6%
range (log_10_ CFU/mL)	(2.00–3.87)	(1.30–2.96)	(0.30–3.86)	(0.00–1.00)	(0.00–1.85)
mean ± SD (log_10_ CFU/mL)	3.11 ± 0.47	1.95 ± 0.60	2.20 ± 1.18	0.14 ± 0.30	0.68 ± 0.73
**HPC 36 °C**					
positive samples (%)	100%	100%	94.14%	27.8%	100%
range (log_10_ CFU/mL)	(3.00–4.00)	(0.56–2.96)	(0.00–3.57)	(0.00–1.04)	(0.30–2.20)
mean ± SD (log_10_ CFU/mL)	3.41 ± 0.37	2.26 ± 0.43	2.30 ± 1.01	0.19 ± 0.36	1.06 ± 0.60
***Pseudomonas aeruginosa***					
positive samples (%)	27.8%	55.6%	27.8%	absent in all samples	absent in all samples
range (log_10_ CFU/100 mL)	(0.00–1.49)	(0.00–1.41)	(0.00–3.00)		
mean ± SD (log_10_ CFU/100 mL)	0.17 ± 0.38	0.55 ± 0.60	0.61 ± 1.05		

As expected, the water from the control unit showed higher microbial loads than those found in the water from the units undergoing either intermittent or continuous disinfection. The differences were statistically significant for both the HPC 22 °C (control *vs.* peroxidic system/deionized water *p <* 0.05; control *vs.* each other treatment *p <* 0.001) and the HPC 36 °C (control *vs.* each other treatment *p <* 0.001). *P. aeruginosa* was detected in 27.8% of the control samples, at concentrations not significantly different from those found in the water of the units undergoing intermittent disinfection, while in the units with continuous disinfection it was always absent. A comparison of the two intermittent disinfection procedures reveals no significant differences between the use of mains water or deionized water on the action of the peroxidic system, for both HPC at 22 °C and 36 °C (mains water *vs* deionized water, *p* > 0.05).

In the continuous disinfection systems the microbial loads were lower than in the units undergoing intermittent disinfection. Both the hydrogen peroxide/silver ions applied to deionized water and the combined reverse osmosis/chlorine bioxide treatment, applied continuously, resulted in a HPC at 22 °C and 36 °C significantly lower than that obtained with each of the intermittent treatments (*p <* 0.001). The stronger action is also evident from the absence of *P. aeruginosa* in all water samples. In comparing the two methods of continuous disinfection only the HPC 36 °C showed significant differences in the mean values: the samples from dental units treated with hydrogen peroxide/silver ions showed lower microbial loads at 36 °C compared to the dental units treated with stabilized ClO_2_ (*p <* 0.05). These results are in agreement with those reported in the literature and confirm, in a public clinical practice setting, the findings obtained in laboratory models [31] and large-scale studies [19] which confirms that continuously applied protocols perform better than those applied intermittently.

Table 2 shows the compliance of the water samples with the standards set by the reference norms for potable water. Both of the methods of continuous disinfection tested in this study (0.02% hydrogen peroxide/silver ions and stabilized chlorine dioxide) made it possible to comply with the CDC guidelines that recommend that the water used for dental treatment meet standards for drinking water set by EPA, AWWA and AWWA. The use of hydrogen peroxide, whose efficacy in the treatment of DUWLs is well documented [8,19,24,26], also allowed compliance with the more restrictive standards for potable water suggested by the European Council Directive 98/83.

**Table 2:** Compliance of samples to water standards.

	Control	Intermittent Disinfection		Continuous Disinfection		
Microbiological indicators	untreated	hydrogen peroxide, peracetic acid 0.26%	hydrogen peroxide, peracetic acid 0.26%		hydrogen peroxide, silver ions 0.02%	stabilized ClO_2_ 0.22%
	mains water n: 18	mains water n: 18	deionized water n: 18		deionized water n: 18	reverse osmosis n: 18
**HPC 22 °C**						
2003 CDC Guidelines (≤500 CFU/mL)	16.7%	66.7%	61.1%		100%	100%
Council Directive 98/83/EC (≤100 CFU/mL)	0	38.9%	38.9%		100%	100%
**HPC 36 °C**						
Council Directive 98/83/EC (≤20 CFU/mL)	0	0	12.4%		100%	72.2%
***Pseudomonas aeruginosa***						
Council Directive 98/83/EC (absence in 100 mL)	72.2%	44.4%	72.2%		100%	100%

### 3.2. Biofilm

**Figure 1:**
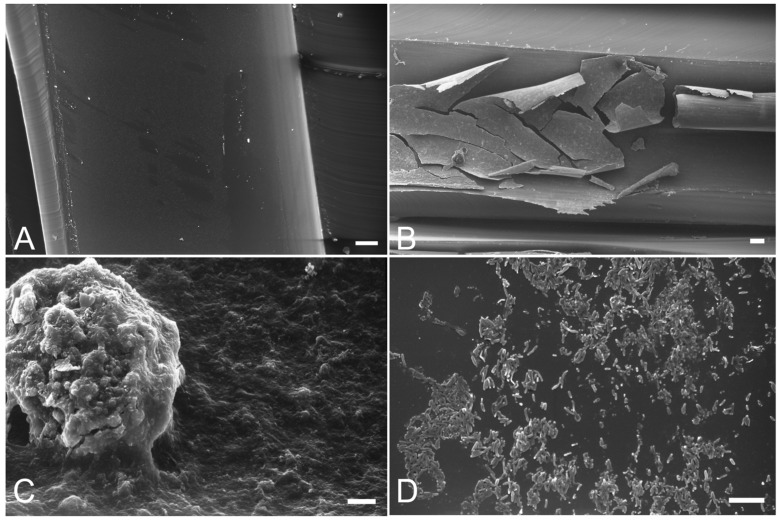
SEM features seen on exposed tube surfaces.

Notes: (A) Control sample of an unused tube. Scale bar = 100μm; (B) Loosely adherent biofilm detached from the tube surface. Scale bar = 100 μm; (C) Bulk, bulging material. Scale bar = 10μm; (D) Clusters of rod-shaped bacteria. Scale bar = 10μm.

Table 3 shows the characteristics of the biofilm at times T_0_ and T_1_. The measurements made on the surfaces of the syringe and turbine tubes have been grouped together since no particular differences were observed. Similarly, the results for the two intermittent treatments and the two continuous treatments are also expressed collectively. At time T_0_ biofilm deposits in the control tubes were so homogeneously distributed along the sampled surfaces that only small areas were left uncoated. All dental unit tubes revealed the presence of very friable biofilm with large detached and fragmented flaps (Figure 1B) and round, bulk aggregates (Figure 1C) possibly representing collections of bacteria, extensive matrix material and salt deposits. Friability, which seems to be associated with greater thickness, could reflect the tendency of the biofilm to fragment leading to a spreading of microorganisms, while the bulk bulging aggregates could represent a mechanism of biofilm propagation. Therefore both friability and bulging contribute to the microbial contamination of water in the DUWLs [8]. At higher magnification SEM analysis of biofilm revealed the presence of long and short rod-shaped bacteria (Figure 1D) which were initially embedded in a thin and loose matrix material and then coalesced into larger aggregates.

At time T_1_ no significant changes were seen in the control samples, while the biofilm characteristics of the treated DUWLs varied considerably, compared to time T_0_ (Table 3). Biofilm thickness significantly decreased both in intermittent and continuous dental tubes (T_0_ *vs*. T_1_ *p <* 0.001), with significant differences between the two protocols (intermittent *vs.* continuous *p <* 0.001). Friability slightly increased in the intermittent dental units (T_1_ *vs*. T_2_ not significant differences) while it was absent in the tubes undergoing continuous disinfection, also as a consequence of the minor thickness found in these samples. Both treatments significantly reduced the presence of bulk deposits from 37% to 3% of surface in dental units using intermittent treatment (*p <* 0.001) and from 65% to 8% of surface in the dental units treated with continuous disinfection (*p <* 0.001). 

**Table 3:** Characteristics of the biofilm in relation to the disinfection protocol.

		Control		Intermittent Disinfection			Continuous Disinfection		
		T_0_	T_1_	T_0_	T_1_	T_0_ *vs.* T_1_	T_0_	T_1_	T_0_ *vs.* T_1_
	surface covered by biofilm <1 μm (%)	15.0	7.5	6.6	46.7		15.3	75.3	
**Thickness**	surface covered by biofilm 1–3 μm (%)	42.5	50.0	46.7	30.8	*p <* 0.001	44.7	23.0	*p <* 0.001
	surface covered by biofilm >3 μm (%)	42.5	42.5	46.7	22.5		40.0	1.7	
**Friability**	surface with detached and fragmented biobilm (%)	70.0	67.5	57.5	67.5	n.s.	63.7	absent	*p <* 0.001
**Bulging**	surface with bulging (%)	65.0	45.0	36.7	3.3	*p <* 0.001	65.0	8.3	*p <* 0.001

Notes: T_0_: before the beginning of the disinfection protocols; T_1_: 3 months after the beginning of the disinfection protocols; ns: not significant.

The differences in biofilm characteristics noted in the intermittent systems at the end of the study, e.g., slight increase in the friability and decrease of bulging, may suggest that intermittent systems, while reducing the capacity of the biofilm to spread on the surfaces, tends to favor detachment, thus enhancing the dispersion of the microorganisms in the water. The continuous treatment systems exerted a more effective action on the biofilm, which was not only notably reduced, but was also not friable and presents less bulging. As previously mentioned, the disinfection systems were installed about three months before the start of the analysis. In this short space of time the treatments were sufficient to radically modify the microbial load patterns and the biofilm characteristics. Although the biofilm has not been completely eliminated (75% of the surface covered by biofilm <1 μm), *P. aeruginosa* was no longer detected in the output water from syringes and turbines.

Some limitations of this study can be considered. The first concerns the HPC culture medium. Most of the studies in the dental literature use R2A agar for the recovery of bacteria from dental units following incubation at 20–22 °C for 7 to 10 days [19,23,29,31]. This medium allows stressed bacteria to recover. In this regard, it is possible that the bacterial counts were underestimated in this study. Secondly, in the present study the microbial contamination has not been associated with some clinical variables such as the number of patients seen per day in every treated unit and the procedures performed. There is evidence to show that amount of clinical use affects effluent microbial levels [4,36].

Another limit of the present study concerns the diversity of the disinfectants used in the intermittent (peroxidic system generating peracetic acid 0.26%) and in the continuous (hydrogen peroxide/silver ions and chlorine dioxide) protocols. Another variable can be found in the water sources (mains water, deionized water or mains water treated with reverse osmosis). The variability in the protocols derives from the choice to focus on public dental care settings, meaning that it was necessary to compare the disinfection systems proposed by the market and chosen by the centers to apply to the dental units, over and above the aims of the study. For this reason it was not entirely possible to perform a correct comparative analysis allowing us to attribute the significant differences obtained with the various treatments to the type of disinfectant used or to the protocol followed for its application. However, it is reasonable to assume, also on the basis of the findings of a recent review by O’Donnell *et al*. [8], that the differences observed are not so much linked to the active product used but rather to the type of protocol applied (intermittent or continuous). In the dental units using intermittent disinfection there is also more chance of human error, since the workers were asked to apply a 10-min disinfection cycle between patients and this operation increases the potential risk of contamination if not correctly performed. The literature reports that non-compliance and technical errors are the most probable causes of failure to properly disinfect DUWLs [31]. In contrast, compliance is favored when using continuous disinfection systems because the equipment is easier to operate, productivity is enhanced and waiting times reduced. The application of the intermittent disinfection system also involves a slight prolongation of the time required between each patient. Practices of decontamination of dental unit normally takes 8–10 min (handpieces and syringes replacement, disinfection of the suction pipe, cleaning the dental unit, *etc*.). The intermittent disinfection requires 5–7 min longer.

## 4. Conclusions

The following conclusions can be drawn from this study:

(1) *S. aureus* and β-haemolytic streptococci were never isolated from the water of any f the units. The risk of contamination by retrograde aspiration was therefore negligible. Also *Legionella* spp and sulphite-reducing *Clostridium* spores were never detected. This satisfactory result could be indirect proof that the recommended preventive measures and good practices were generally applied.
(2) The high microbial loads found in the untreated control dental unit confirm what has already been widely shown in the literature and highlight the necessity of supplying the DUWLs with disinfection systems. The application of the various disinfection protocols brought about statistically significant reductions in the microbial loads compared to the control, but only continuous disinfection ensured the total abatement of *P. aeruginosa*.
(3) Biofilm was present to a greater extent and in thicker and more friable layers in the syringe and turbine tubes of the control dental unit than in those undergoing intermittent disinfection and, even more so, continuous disinfection systems. Also the bulk aggregates were significantly more present in the tubes of the control dental unit.

These findings show that, under the conditions used in the study, the products used for continuous treatment of dental unit waterlines show better results than those used for intermittent treatment. 
